# Efficacy and safety of the Chinese herbal medicine Xiao Yao San for treating anxiety: a systematic review with meta-analysis and trial sequential analysis

**DOI:** 10.3389/fphar.2023.1169292

**Published:** 2023-10-12

**Authors:** Yifan Wang, Xiaofeng Chen, Wei Wei, Yiyun Ding, Rongjuan Guo, Jia Xing, Jialin Wang

**Affiliations:** ^1^ Dongfang Hospital, Beijing University of Chinese Medicine, Beijing, China; ^2^ The First Affiliated Hospital, Yunnan University of Chinese Medicine, Kunming, China; ^3^ Psychosomatic Department, Dongfang Hospital, Beijing University of Chinese Medicine, Beijing, China; ^4^ Rehabilitation Department, Dongfang Hospital, Beijing University of Chinese Medicine, Beijing, China

**Keywords:** anxiety disorder, Chinese herbal medicine, Xiao Yao San, systematic review, meta analysis, trial sequential analysis

## Abstract

**Introduction:** The effectiveness and safety of the Chinese herbal medicine (CHM) Xiao Yao San (XYS) used for treating anxiety disorders are still unknown. Thus, we conducted this systematic review with meta-analysis and trial sequential analysis (TSA) to determine its safety and efficacy.

**Methods:** We searched 12 databases for relevant studies from the inception of each database till 10 August 2023. We selected randomized controlled trials to compare the efficacy and safety of XYS (including XYS only and XYS + anxiolytics) to those of anxiolytics in patients with anxiety.

**Results:** We found 14 trials with 1,256 patients in total that met the requirements for inclusion. We assessed the majority of studies (8 out of 14) as being at high risk of bias; 6 were assessed as having a moderate risk of bias. Three trials compared oral XYS to anxiolytic medication, and 11 trials compared oral XYS plus anxiolytics to anxiolytic treatment alone. The pooled results showed that the efficacy of treatment in the XYS + anxiolytics groups was significantly higher than that of the anxiolytics alone group (RR = 1.19; 95% CI: [1.13, 1.26]; *p* < 0.00001; I^2^ = 0) and the adverse event rates in the XYS + anxiolytics groups were significantly lower than those in the anxiolytics alone group (RR = 0.44; 95% CI: [0.28, 0.82]; *p* = 0.001 < 0.05; I^2^ = 13). The efficacy of treatment in the XYS alone groups was also significantly higher than that of the anxiolytics alone groups (RR = 5.41; 95% CI: [2.23, 13.11]; *p* < 0.0001; I^2^ = 0). However, there was no statistical difference between the adverse events of the XYS alone group and the anxiolytics alone group, although the incidence of adverse events in the XYS alone group was lower than that in the anxiolytics alone group. The results of the TSA confirmed the above findings.

**Conclusion:** The use of XYS combined with anxiolytics for treating anxiety was found to be safe and effective. However, although XYS alone is effective in the treatment of anxiety disorder, more large-scale research is needed to investigate adverse events.

**Systematic Review Registration:** https://www.crd.york.ac.uk/PROSPERO/display_record.php?RecordID=350358, identifier CRD42022350358.

## 1 Introduction

Anxiety disorder is a common psychiatric disorder, which has become a major public global health problem. Anxiety disorder has high morbidity and ranked 24th in terms of disability-adjusted life years (DALYs) in the global prevalence estimates and disability weights in 2019 (Collaborators, 2022). Since the onset of the COVID-19 pandemic, the prevalence of anxiety disorders has increased significantly. In 2020, the prevalence of anxiety disorders worldwide was 26% (Collaborators Covid- Mental Disorders, 2021). It was estimated that due to the COVID-19 pandemic, around 76 million new cases of anxiety disorder would arise globally (Collaborators Covid- Mental Disorders, 2021).

Anxiety disorder is characterized by excessive anxiety and worry. It affects daily physical, psychological, and social functions, and its pathophysiological mechanism is not clear. Studies on the pathophysiological mechanism of anxiety involve genetics, basic neuroscience, transformation research of functional MRI, etc. (Penninx et al., 2021).

Psychotherapy, drug therapy, and combination therapy are recommended for the clinical treatment of anxiety disorder (Slee et al., 2019; Penninx et al., 2021). Psychotherapy is the first-line treatment for anxiety disorder, but popularizing it in clinical practice was difficult due to the limited resources of psychotherapy, the lack of qualification and experience of clinicians, the time required to show effect, and the cost to patients (Moritz et al., 2019). Drug treatment is often regarded as the first choice for treating anxiety disorder, as it is cheap and accessible (Slee et al., 2019). The drugs used for treating anxiety disorders include antidepressants, benzodiazepines (BZs), other anxiolytics (buspirone, agomelatine, pregabalin, and quetiapine), etc. (Slee et al., 2019).

Antidepressants, including selective serotonin reuptake inhibitors (SSRIs), norepinephrine reuptake inhibitors (SNRIs), and tricyclic antidepressants (TCAs), are often used for treating anxiety disorders. However, at the beginning of treatment, antidepressants may have adverse reactions, such as increased anxiety symptoms, irritability, insomnia, dizziness, and nausea. The onset of anti-anxiety effects has a latency of 2–6 weeks (Slee et al., 2019). Discontinuation of anxiolytics may cause withdrawal symptoms, and adverse events such as sexual dysfunction and gastrointestinal bleeding might occur, which can affect the compliance of patients with medication (Baldwin et al., 2011). Benzodiazepines can produce anti-anxiety effects within 30–60 min, but may affect cognitive function, produce dizziness, prolong reaction time, and other adverse reactions. Short-term administration of benzodiazepines for treating anxiety disorders is usually safe and effective, and maintenance treatment is needed to weigh the risks and benefits (Bandelow et al., 2022). Other anxiolytics (buspirone, agomelatine, pregabalin, and quetiapine) have adverse reactions, such as hepatotoxicity, weight gain, and discontinuation symptoms, which might reduce the patient’s medication compliance (Baldwin et al., 2011; Freiesleben and Furczyk, 2015; Maneeton et al., 2016).

Based on the limitations of clinical treatment methods, as prospective therapeutic and preventative strategies for anxiety, complementary and alternative medicine (CAM) and traditional Chinese medicine (TCM) have received attention. XYS is a traditional Chinese medicine prescription, first recorded in the “Taiping Huimin Heji Jufang”. The eight Chinese herbs that are found in XYS are Chai Hu (Radix Bupleuri), Bai Shao (Radix Paeoniae alba), Dang Gui (Radix Angelicae sinensis), Bai Zhu (Atractylodes Ovata), Fu Ling (Poria Cocos), mint (Herba Menthae), licorice (Radix Glycyrrhizae), and Stewed ginger (Rhizoma Zingiberis Recens). All significant XYS components, including paeoniflorin, quercetin, luteolin, farnesin, aloe emodin, glyasperin C, and kaempferol, were identified by the UPLC-Q-TOF/MS analysis. The primary chemicals were flavonoids (Yuan et al., 2020). For thousands of years, XYS has been used to treat mental illnesses and functional gastrointestinal disorders (Liu et al., 2021; Zhu et al., 2022). Several studies have also shown that using XYS to treat anxiety results in anxiolytic activity. Additionally, the administration of XYS to treat anxiety was found to cause anxiolytic activity (Cao et al., 2016). Some animal studies have shown that XYS can reduce the increase in synuclein and corticosterone caused by chronic stress. It can also downregulate protein phosphatase 2A (PP2A) in the hippocampus to produce anti-anxiety and neuroprotective effects (Cao et al., 2016).

XYS is frequently used for treating mental illnesses. Although XYS is frequently used to treat depression (Yuan et al., 2020; Liu et al., 2021), its effectiveness in treating anxiety disorders is still unclear (Ding et al., 2013; Wang, 2014). In several randomized controlled trials (RCTs), XYS had positive curative effects on anxiety disorder (Xiong and Song, 2019; Zhou et al., 2021). However, more evidence is needed to confirm the safety and effectiveness of XYS in the treatment of anxiety disorders. Therefore, we performed a systematic review through meta-analysis to compile the evidence for the treatment of anxiety disorder with XYS to objectively assess the literature. This review might serve as a reference for future therapeutic medication and clinical research. We also performed trial sequential analysis (TSA) to estimate the sample size.

## 2 Materials and methods

The literature review did not require ethical approval. The Preferred Reporting Items for systematic reviews and meta-analyses (PRISMA) were followed in the planning, execution, and reporting of the findings of this study (Page et al.). The PRISMA checklist is available in the Supplementary Material. The protocol is registered in PROSPERO under the registration number CRD42022350358 (Gao et al., 2021).

### 2.1 Literature search strategy

In total, 12 databases, including Embase, MEDLINE, Cochrane Library, PsycINFO, The Allied and Complementary Medicine Database (AMED), PubMed, the Web of Science, Scopus, the China National Knowledge Infrastructure database (CNKI), Wanfang Data, the China Biomedical Medicine database (SinoMed), and VIP Chinese Medical Journal Database(CMJD), were searched from their inception to 10 August 2023. Keywords or forms of keywords used in the database searches included Anxiety, anxiety disorders, xiaoyao san, xiaoyao formula, kami-shoyo-san, gamisoyo-san, soyo-san, kamo-soyo-san. A copy of the search strategies used for each database is shown in Supplementary Material. To obtain more information, reference materials, and conference proceedings were personally reviewed. When necessary, we contacted the associated authors to obtain any material that was omitted or incomplete. Chinese characters were used to search for terms in the electronic databases in China.

### 2.2 Inclusion criteria

The studies considered for additional analysis were based on the following criteria: 1) All studies were randomized controlled trials (RCTs). 2) Participants: The International Classification of Diseases, the Diagnostic and Statistical Manual of Mental Disorders, and the Chinese Classification of Mental Disorder require that all enrolled patients meet specific diagnostic criteria for anxiety; 3) Patients in the intervention groups received either XYS (no restrictions on dosage, formula, or dosage form) or XYS combined with anxiolytics, whereas, the control group received only anxiolytics; 4) Results: At least one important outcome with valid and readily accessible data.

### 2.3 Exclusion criteria

Cases that matched the criteria below were disqualified: 1) Anxiety disorder along with other conditions, including Parkinson’s disease, dermatitis, cerebral infarction, etc. 2) Insufficient information on the medication used in the experiment; 3) Experiments in which the baseline data for the observation group and the control group were distinct and incomparable; 4) The treatment group was administered Chinese patent medications other than XYS; 5) Incomplete information or mistakes; 6) No extractable outcome indicators were available; 7) Repetitive publication of studies retaining one article; 8) Non-journal papers.

### 2.4 Outcome measures

The Clinical Efficacy Rate, the score on the Hamilton Anxiety Rating Scale (HAMA), and the Adverse Effects Rate were the main outcomes. The secondary outcomes included the SAS score, the PSQI score, and the TESS score.

### 2.5 Data extraction and quality assessment

Using the inclusion and exclusion criteria mentioned above, two researchers, Yifan Wang and Xiaofeng Chen, independently downloaded articles from the databases and extracted the data using the template data extraction form. Basic study parameters (diagnosis criteria, research duration, and total sample size), interventions (drugs, dosages, formulae, and dosage form), and outcomes were included in the data that were taken from each report. The baseline patient characteristics included gender distribution, disease course, and age. Two researchers cross-checked and consulted with each other and with another researcher (Prof. Guo), or, if necessary, the authors of the studies were contacted to settle disagreements in the included papers.

The Cochrane Risk of Bias tool (Higgins et al., 2022) was used to assess the methodological quality of the selected studies. To assess the quality of evidence and interpret the results, we applied six RoB criteria (random sequence generation, allocation concealment, blinding of participants, blinding of outcome assessors, incomplete outcome data, and selective outcome reporting) to categorize each trial. A trial would be categorized as having a low risk of bias if none of the domains above were rated as high risk of bias, and two or fewer were classified as unclear risk. A trial would be categorized as having a moderate risk of bias if one domain was rated as high risk of bias, one or fewer domains were classified as unclear risk, or no domains were rated as high risk of bias but three or fewer were classified as unclear risk. All other cases were considered to fall under the category of high risk of bias (Sbidian et al., 2023). Two researchers, Jialin Wang and Jia Xing, independently assessed the risk of bias. Prof. Guo, another author, was also invited to settle any disagreements.

### 2.6 Data synthesis and statistical analysis

The risk ratio (RR) and mean differences (MD) were used in the meta-analysis, which was conducted using RevMan 5.4.1 (The Cochrane Collaboration). The overall mean differences and the related 95% confidence intervals were calculated using the random-effects model when heterogeneity (I^2^ > 50%) was found between various treatment groups in the included studies. Otherwise, the fixed-effects model was used to perform the calculations. All differences were considered to be statistically significant at *p* < 0.05. Different studies had different definitions of effective rate, and we speculated that significant clinical heterogeneity was present. Hence, we used the random effects model (Higgins et al., 2022). Publication bias was examined by conducting funnel plot analyses if the number of studies was ≥10. Sensitivity analyses were conducted to examine the impact of individual studies on the overall effect estimate.

### 2.7 Trial sequential analysis

To reduce the possibility of a false-positive or false-negative conclusion, the Copenhagen Trial Unit, Centre for Clinical Intervention used TSA Version 0.9.5.10 Beta to perform the Trial Sequential Analysis (TSA) (Pogue and Yusuf, 1997; Brok et al., 2008). The models for this TSA set the type I error (α) at 0.05 and a power of 80% (two-sided) for all outcomes. The sequential monitory boundary varied each analysis in this TSA, and the usual significance boundary was −1.96 to 1.96.

## 3 Results

### 3.1 Study selection

**FIGURE 1:**
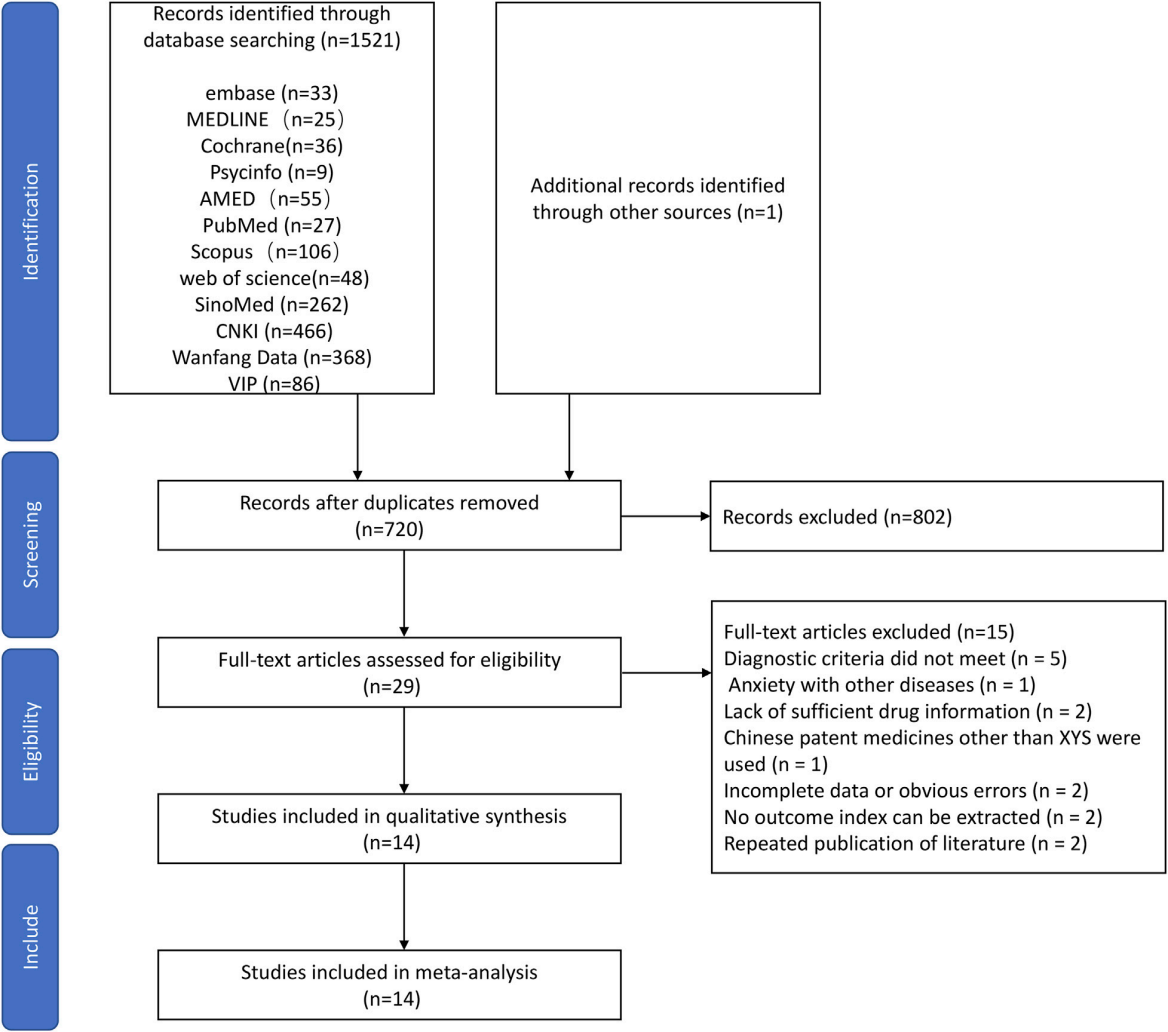
A flow diagram of the screening process used to select the articles in this study.

Using the predefined search strategy, we obtained 1,522 studies in total. After removing duplicates, 720 studies remained. After excluding 691 articles by screening the title and abstract, 29 articles were left for a full-text analysis of the duplicates. In total, 15 papers were rejected. The diagnostic standards were not met in five studies; various diseases and anxiety were recorded in 1 studies; inadequate medication information was recorded in two studies; other Chinese patent drugs besides XYS were administered to the treatment group in one study; incomplete information or glaring mistakes were found in two studies; no outcome index could be retrieved from two studies; repeated publication of data in two study. Finally, 14 RCTs (Wang et al., 2010; Han et al., 2011; Ding et al., 2013; Lv et al., 2013; Wang, 2014; Zhu, 2015; Chen and Du, 2016; LI and Qin, 2016; Deng et al., 2017; Li et al., 2017; Shi, 2018; Xiong and Song, 2019; Zhou et al., 2021; Li et al., 2022) were eligible for the study. The screening process of the systematic review is shown in Figure 1.

The selected RCTs included 1,256 people, 633 of whom were in the intervention groups, and 623 were in the control groups. The baseline information for the treatment and control groups was similar. Anxiolytics were used in the treatment plans for the individuals in the control groups. Chinese herbal medicine XYS or XYS combined with anxiolytic drugs was administered to the patients in the treatment groups. The data on the characteristics of those studies are summarized in Table 1.

**TABLE 1:** Characteristics of the included studies.

Study ID	Diagnostic criteria	Sample size		Course of disease (month)		Mean age (year)		Male Proportion(Male%)		Intervention		Course of treatment (week)	Outcomes
		Trial	Control	Trial	Control	Trial	Control	Trial	Control	Trial	Control		
Deng et al. (2017)	CCMD-3	59	57	18.18 ±13.56	17.93 ±12.84	43.81 ±10.23	44.53 ±11.65	44.07%	52.63%	XYS + C	Paroxetine	4	CER, HAMA-14, AER
Ding et al. (2013)	CCMD-3	37	38	25.15 ±10.84(day)	27.21 ±6.72(day)	29.46 ±6.82	27.62 ±4.24	NR	NR	XYS + C	Buspirone + Lorazepam	8	CER, HAMA-14, SAS
Han et al. (2011)	CCMD-2R	40	40	18–155	12–168	22–77	23–76	42.50%	37.50%	XYS + C	Deanxit	12	CER, HAMA-14
Li and Qin (2016)	CCMD-3	32	32	57.6 ±28.8	58.8 ±30.0	41.6 ±7.8	42.1 ±7.2	46.88%	43.75%	XYS + C	Buspirone	6	CER, HAMA-14, TESS
Li et al. (2022)	CCMD-3	40	40	≥1	≥1	48.50 ± 1.25	46.00 ± 1.06	42.50%	40.00%	XYS + C	Buspirone	6	CER, HAMA-14, AER
Lv et al. (2013)	CCMD-3	36	35	12.3 ±NR	11.2 ±NR	35.5 ±NR	34.4 ±NR	33.30%	40.00%	XYS + C	Deanxit + psychological counseling	8	CER, HAMA-14
Wang (2014)	CCMD-3	30	30	18–204	12–180	21–75	23–72	33.30%	40.00%	XYS + C	Paroxetine	8	CER, HAMA-14
Wang et al. (2010)	CCMD-3	30	30	NR	NR	48 ±NR	51 ±NR	53.33%	50.00%	XYS + C	Deanxit	6	CER, HAMA-14
Xiong and Song. (2019)	CCMD-3	100	100	NR	NR	57.8 ±6.5	55.4 ±6.2	65.00%	62.00%	XYS + C	Deanxit(+Tandospirone for severe patients)	8	CER, HAMA-14, AER, PSQI
Zhou et al. (2019)	CCMD-3	35	35	4.37 ±2.52	4.37 ±2.52	44.86 ±10.81	46.29 ±12.28	34.29%	28.57%	XYS + C	Mirtazapine	4	CER, HAMA-14, AER, PSQI
Zhu (2015)	CCMD-3	28	28	61.68 ±29.52	61.68 ±29.52	42.86 ±14.12	42.86 ±14.12	NR	NR	XYS + C	Buspirone	6	CER, HAMA-14, TESS
Chen and (2016)	DSM-IV	78	76	23.28 ±5.72	23.12 ±3.37	43.65 ±8.01	43.93 ±10.54	49.38%	51.28%	XYS	Deanxit	6	CER, HAMA-14, SAS
Li et al. (2017)	CCMD-3	48	42	17.86 ±7.34	17.76 ±7.84	55.15 ±11.50	54.71 ±10.77	39.58%	42.86%	XYS	Lorazepam	4	CER, HAMA-14, AER, TESS
Shi (2018)	ICD-10	40	40	NR	NR	33.4 ±12.8	34.3 ±12.4	47.50%	45.00%	XYS	Deanxit	4	CER, HAMA-14

Abbreviations: AER, adverse events rates; CCMD, Chinese classification of mental disorders; CER, clinical efficacy rates; DSM, diagnostic and statistical manual of mental disorders; ER, efficacy rates; HAMA-14, Hamilton Anxiety Scale-14; ICD, international statistical classification of diseases and related health problems; NR, not reported; PSQI, pittsburgh sleep quality index; SAS, Self-Rating Anxiety Scale; TESS, treatment emergent symptom scale.

### 3.2 Risk of bias assessment

**FIGURE 2:**
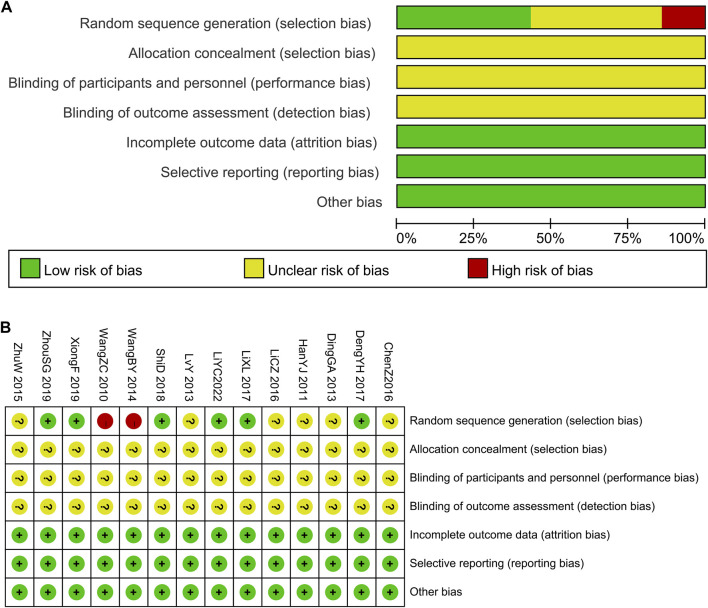
The risk of methodological bias in the included studies. **(A)** Risk of bias graph. **(B)** Risk of bias summary.

Six studies (Deng et al., 2017; Li et al., 2017; Shi, 2018; Xiong and Song, 2019; Zhou et al., 2021; Li et al., 2022) explained the randomization procedure (random number table). The patients were divided into groups by parity order of enrollment (Wang, 2014), and it was considered as high risk. Additionally, the other studies did not outline their randomization approach completely. Uncertainties were recorded regarding allocation concealment and outcome assessment blinding. These studies were not double-blinded. Complete data and reliable results were available for all articles included, as stated in the section on techniques. No differences occurred in the baseline data of these studies. The results of the risk of bias assessment are presented in Figure 2.

In terms of overall bias risk, six studies (Deng et al., 2017; Li et al., 2017; Shi, 2018; Zhou et al., 2021; Li et al., 2022) were considered to have a moderate risk of bias, while eight studies (Wang et al., 2010; Han et al., 2011; Ding et al., 2013; Lv et al., 2013; Wang, 2014; Zhu, 2015; Chen and Du, 2016; LI and Qin, 2016) were considered to have a high risk of bias.

### 3.3 Outcome measures

The outcomes of treatment with XYS combined with anxiolytics were compared to the outcomes of treatment with anxiolytics in 11 of 14 studies, whereas three studies compared the outcomes of treatment with oral XYS alone to those of treatment with anxiolytics.

The HAMA scores and clinical symptoms were the main means to gauge the clinical effectiveness. Eight studies (Wang et al., 2010; Ding et al., 2013; Wang, 2014; Zhu, 2015; LI and Qin, 2016; Zhou et al., 2021; Li et al., 2022) reported the clinical efficacy rate by a HAMA score reduction rate >25%. A HAMA score reduction rate of >30% was reported as the clinical effectiveness rate in one study (Chen and Du, 2016).

In a study (Deng et al., 2017), the clinical efficacy rate was calculated using a HAMA score drop rate of >30% and combined with the clinical symptoms. Using the clinical symptoms, two studies (Han et al., 2011; Lv et al., 2013) reported the clinical effectiveness rate. A decrease in the Chinese medicine syndrome scale score of >30%, along with the clinical symptoms, was used in one study (Li et al., 2017) to determine the clinical effectiveness rate. One study (Shi, 2018) found that a reduction rate of >25% in the Chinese medicine syndrome scale score indicated therapeutic efficacy.

#### 3.3.1 Comparison of XYS combined with anxiolytics to anxiolytics alone

##### 3.3.1.1 Clinical efficacy rates

**FIGURE 3:**
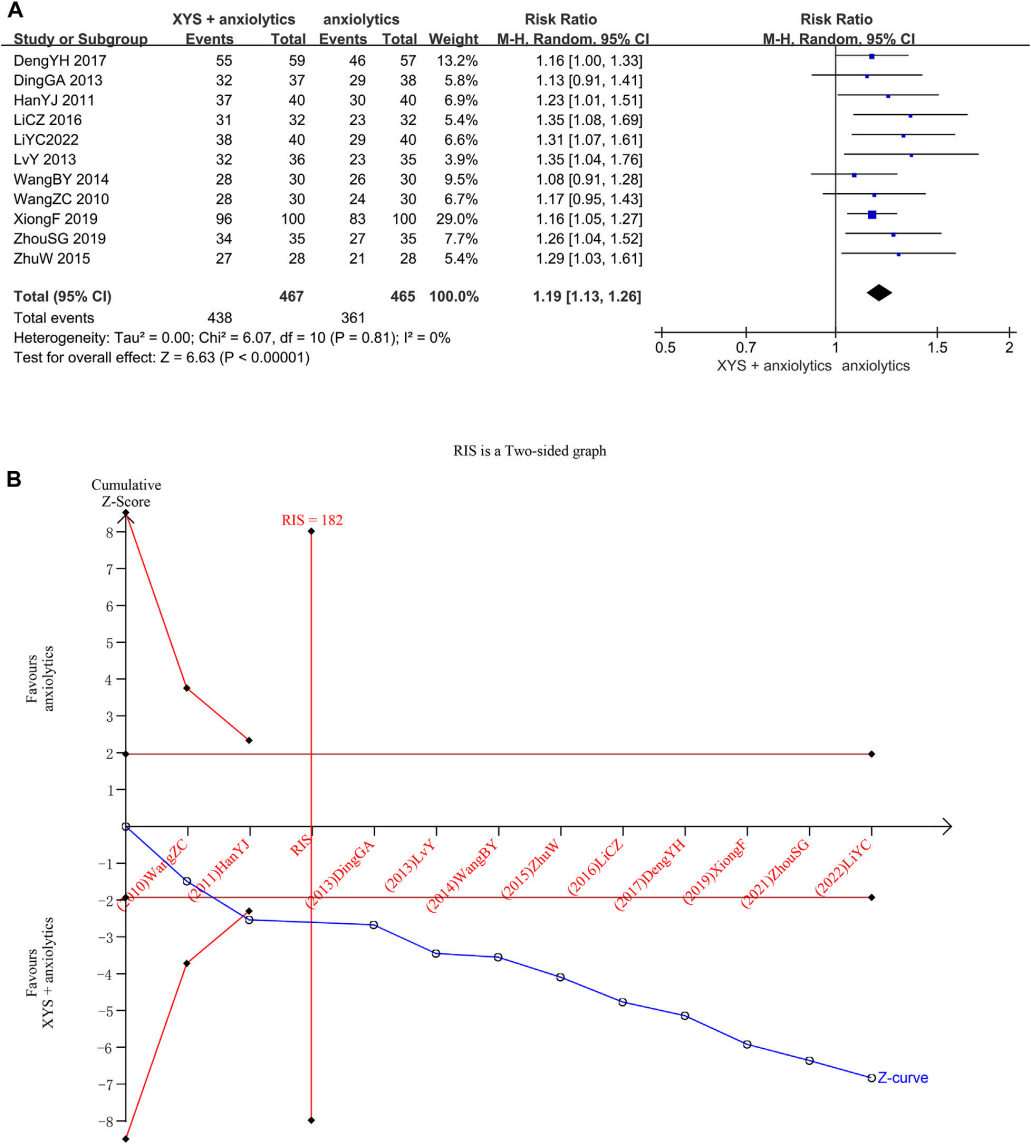
Data analysis of clinical efficacy rates (XYS combined with anxiolytics vs. anxiolytics alone). **(A)** Meta-analysis. **(B)** Trial sequential analysis. The blue line represents the cumulative number of cases in the meta-analysis, also called the “Z-curve”. The horizontal straight red line shows the conventional test boundary, which is a traditional significant horizontal line (*α* = 0.05). The twisted red line represents the trial sequential monitoring boundary, which is an interface curve formed by correcting the random error generated by the meta-analysis. The vertical, straight red line represents the required information size (RIS) and refers to the number of cases required for meta-analysis to obtain statistically significant differences. The cumulative Z-curve surpassed the point of no return and increased to the necessary information size.

In 11 studies (932 patients), the clinical efficacy rates were reported. Between different investigations, heterogeneity was not detected (Chi^2^ = 6.07, *p* = 0. 81, I^2^ = 0%). The efficacy achieved in the treatment groups was considerably higher than that in the control groups, determined by the results of the random effects model (RR = 1.19, 95% CI: [1. 13, 1. 26], *p* < 0.00001; Figure 3A). Publishing bias was not found (the Egger’s test yielded a *p*-value of 0.054.), as determined by the symmetry of the funnel plot (Supplementary Figure S1).

We confirmed the reliability of the meta-analysis through TSA. The cumulative Z-curve crossed the trial sequential boundary, and the traditional boundary after the second study was included, indicating a low probability of false positives. When the third study was included, the cumulative sample size crossed the required information size (RIS) (182). The results of TSA matched those of the meta-analysis, suggesting that compared to the effects of anxiolytics alone, the effects of XYS + anxiolytics significantly improved the anxiety symptoms of patients with emotional disorders; the results were stable, and the evidence was reliable (Figure 3B).

##### 3.3.1.2 HAMA scores

**FIGURE 4:**
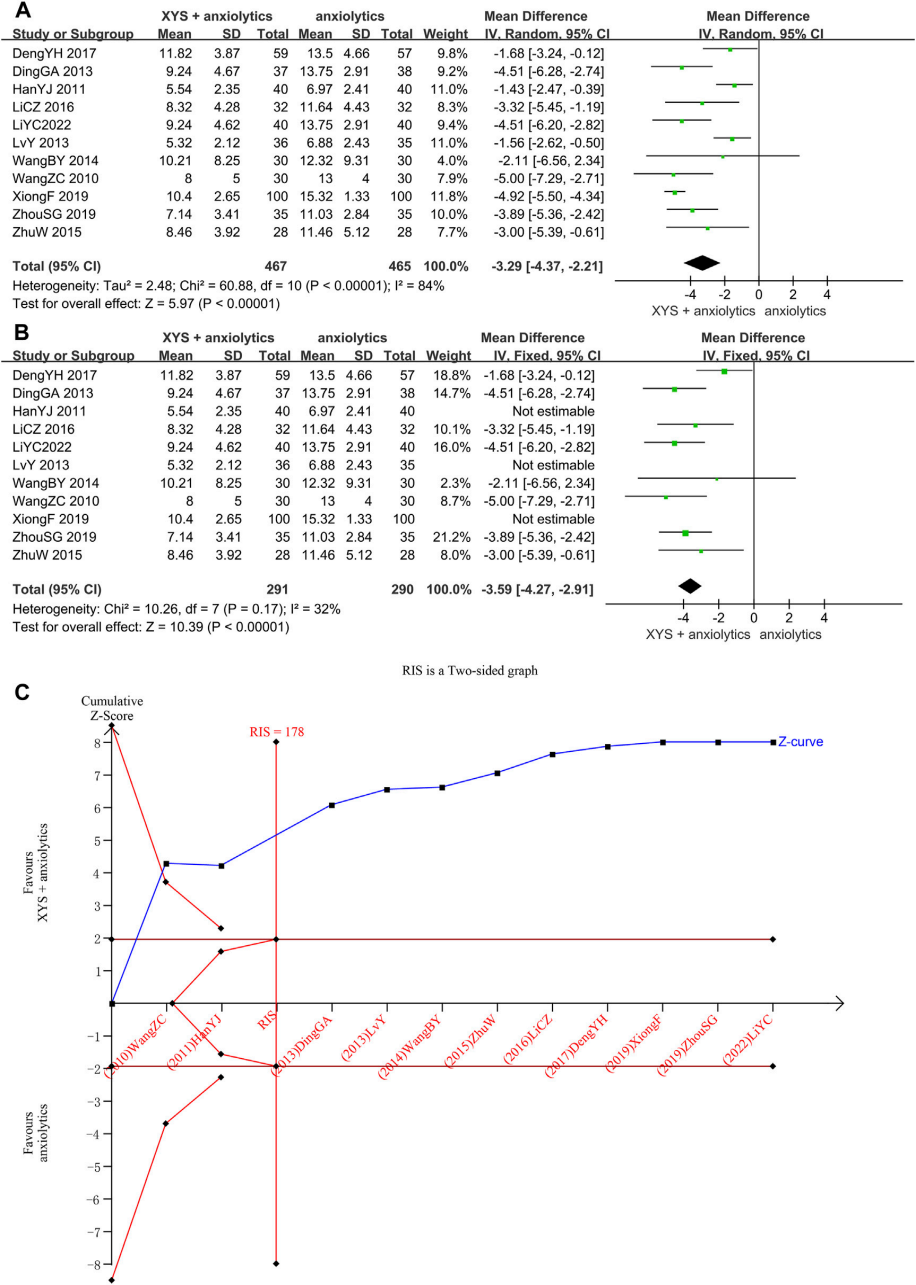
Data analysis of the HAMA-14 scores (XYS combined with anxiolytics vs. anxiolytics alone). **(A)** Before eliminating sources of heterogeneity. **(B)** After eliminating sources of heterogeneity. **(C)** Trial sequential analysis. The annotations of the red line and blue line in TSA are the same as those in Figure 3.

The HAMA scores were provided in 11 studies (Wang et al., 2010; Han et al., 2011; Ding et al., 2013; Lv et al., 2013; Wang, 2014; Zhu, 2015; LI and Qin, 2016; Deng et al., 2017; Xiong and Song, 2019; Zhou et al., 2021; Li et al., 2022), with significant heterogeneity among these studies (*p* < 0.00001, I^2^ = 84%). The random-effects meta-analysis estimated that the HAMA scores were significantly lower in the XYS combined with the anxiolytics group relative to the anxiolytics group ([MD = −3.29, 95% CI: −4.37, −2.21, *p* < 0.00001]; Figure 4A). A visual analysis of the funnel plot revealed some level of publishing bias (Supplementary Figure S2). Nevertheless, Egger’s test failed to reach statistical significance (*p* = 0.372). We tried reducing heterogeneity by classifying by the course of disease and interventions but failed. The sensitivity analysis showed that three studies (Han et al., 2011; Lv et al., 2013) were the sources of heterogeneity. One study (Han et al., 2011) had the longest course of treatment (12 weeks) and the lowest HAMA score before treatment. The HAMA score before treatment was 14.97 ±2.79 in the treatment group and 14.83 ±2.61 in the control group. In contrast, the lowest score in other studies was 19.37 ±4.50 in the treatment group and 20.12 ±5.29 in the control group (Zhou et al., 2021). In one study (Lv et al., 2013), the patients in the treatment group and the control group were treated via psychological counseling. In one study (Xiong and Song, 2019), the administration of oral buspirone citrate in patients with severe anxiety caused heterogeneity. A meta-analysis of the other seven studies suggested that oral XYS combined with anxiolytics treatment has higher efficacy than anxiolytics treatment alone as a means of lowering anxiety disorder patient HAMA with low heterogeneity ([MD = −3.59, 95% CI: −4.27, −2.91, *p* < 0.00001]; I^2^ = 32%; Figure 4B).

Through TSA, we confirmed the validity of the meta-analysis. For the first study, the cumulative Z-curve crossed the traditional and trial sequential boundaries, indicating a low likelihood of false positives. The total sample size reached the RIS (178) when the third study was added. The data were reliable, and the TSA results were stable, which showed that XYS combined with anxiolytics could considerably lower the HAMA score of patients with anxiety symptoms compared to anxiolytics (Figure 4C).

##### 3.3.1.3 SAS scores

One RCT study reported the SAS scores (Ding et al., 2013). As we could not conduct a meta-analysis and trial sequential analysis, descriptive analysis was performed, which showed significantly lower SAS scores in the XYS combined with the anxiolytics group relative to the anxiolytics group ([MD = −4.56, 95% CI: −7.19, −1.93]), *p* = 0.0007).

##### 3.3.1.4 PSQI scores

**FIGURE 5:**
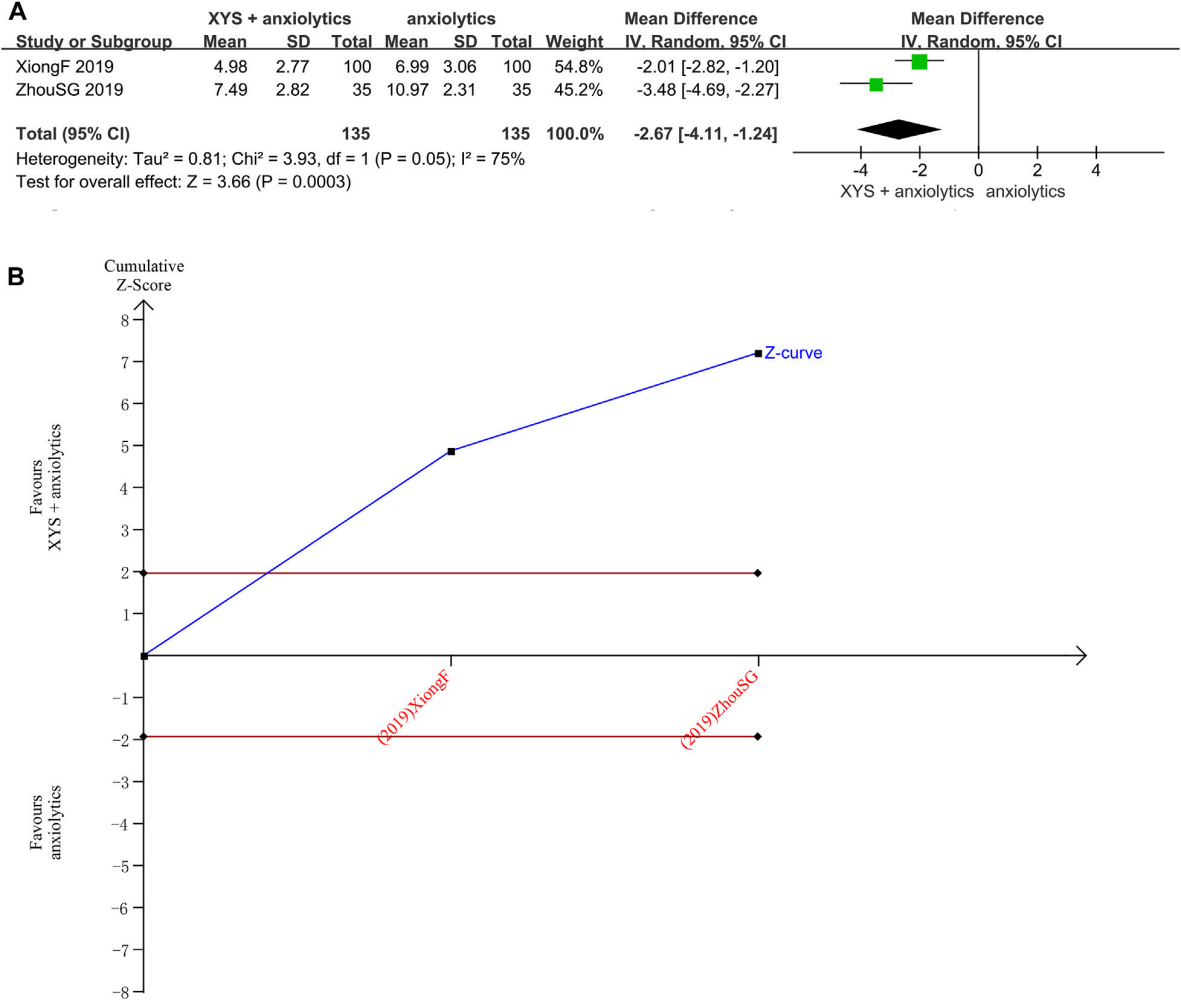
Data analysis of the PSQI scores (XYS combined with anxiolytics vs. anxiolytics alone). **(A)** Meta-analysis. **(B)** Trial sequential analysis. The annotations of the red line and blue line in TSA are the same as those in Figure 3.

The PSQI scores were reported in two studies (Zhou et al., 2021) with significant heterogeneity between those studies (*p* = 0.05, I^2^ = 75%). The random-effects meta-analysis estimated that the PSQI scores were significantly lower in the treatment group relative to that in the control group ([MD = −2.67, 95% CI: −4.11, −1.24, *p* < 0.00001]; Figure 5A). The source of heterogeneity was probably the administration of tandospirone to patients with severe anxiety (Xiong and Song, 2019).

The TSA analysis for PSQI scores showed that the cumulative Z-curve exceeded the traditional significance boundary, but the sequential monitoring boundary was not rendered because the first information fraction exceeded 100%, which indicated that the first study had sufficient statistical power for a meta-analysis. The TSA suggested that compared to the anxiolytics, XYS combined with anxiolytics significantly decreased the PSQI score of patients with anxiety symptoms, the results were stable, and the evidence was reliable (Figure 5B).

##### 3.3.1.5 Adverse events rates

**FIGURE 6:**
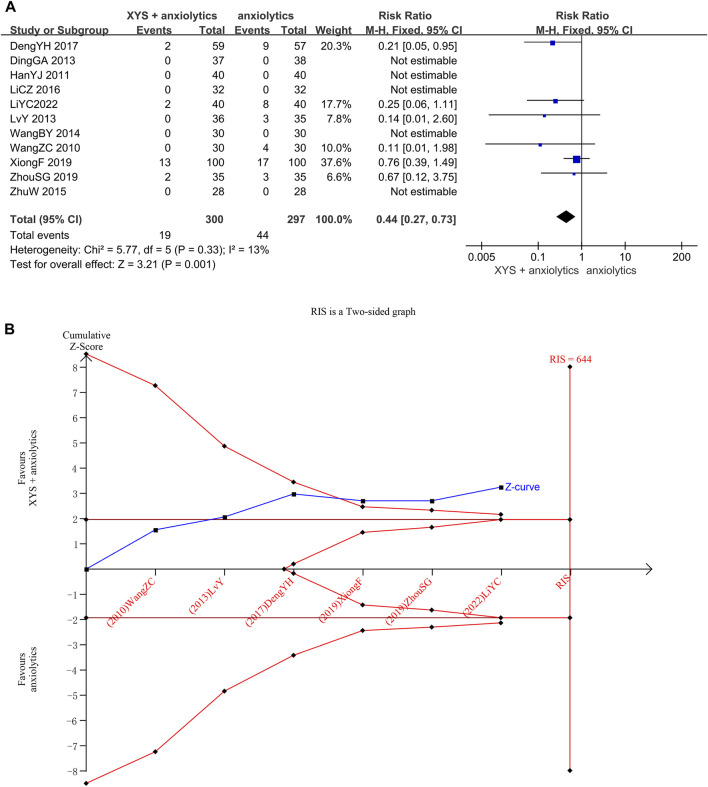
Data analysis of Adverse Events Rates (XYS combined with anxiolytics vs. anxiolytics alone). **(A)** Meta-analysis. **(B)** Trial sequential analysis. The annotations of the red line and blue line in TSA are the same as those in Figure 3.

Adverse event rates were reported in Six studies (Wang et al., 2010; Lv et al., 2013; Deng et al., 2017; Zhou et al., 2021; Li et al., 2022). No heterogeneity was detected when evaluating these studies (*p* = 0.33, I^2^ = 13%), and the results were thus analyzed using a fixed-effects model. The pooled meta-analysis showed that the adverse event rates in the treatment group were significantly lower than those in the control group ([RR = 0.44, 95% CI: 0.27, 0.73, *p* = 0.001]; Figure 6A).

We confirmed the reliability of the meta-analysis through TSA. The cumulative Z-curve crossed the trial sequential boundary and the traditional boundary in the second study, indicating a low probability of a false positive. Although the accumulated information did not reach the expected value, no more tests were needed to reach a positive conclusion in advance. The results of TSA matched those of the meta-analysis, suggesting that compared to anxiolytics treatment, the XYS combined with anxiolytics treatment significantly decreased the adverse reactions of patients with anxiety disorder; the results were stable, and the evidence was reliable (Figure 6B).

##### 3.3.1.6 TESS scores

**FIGURE 7:**
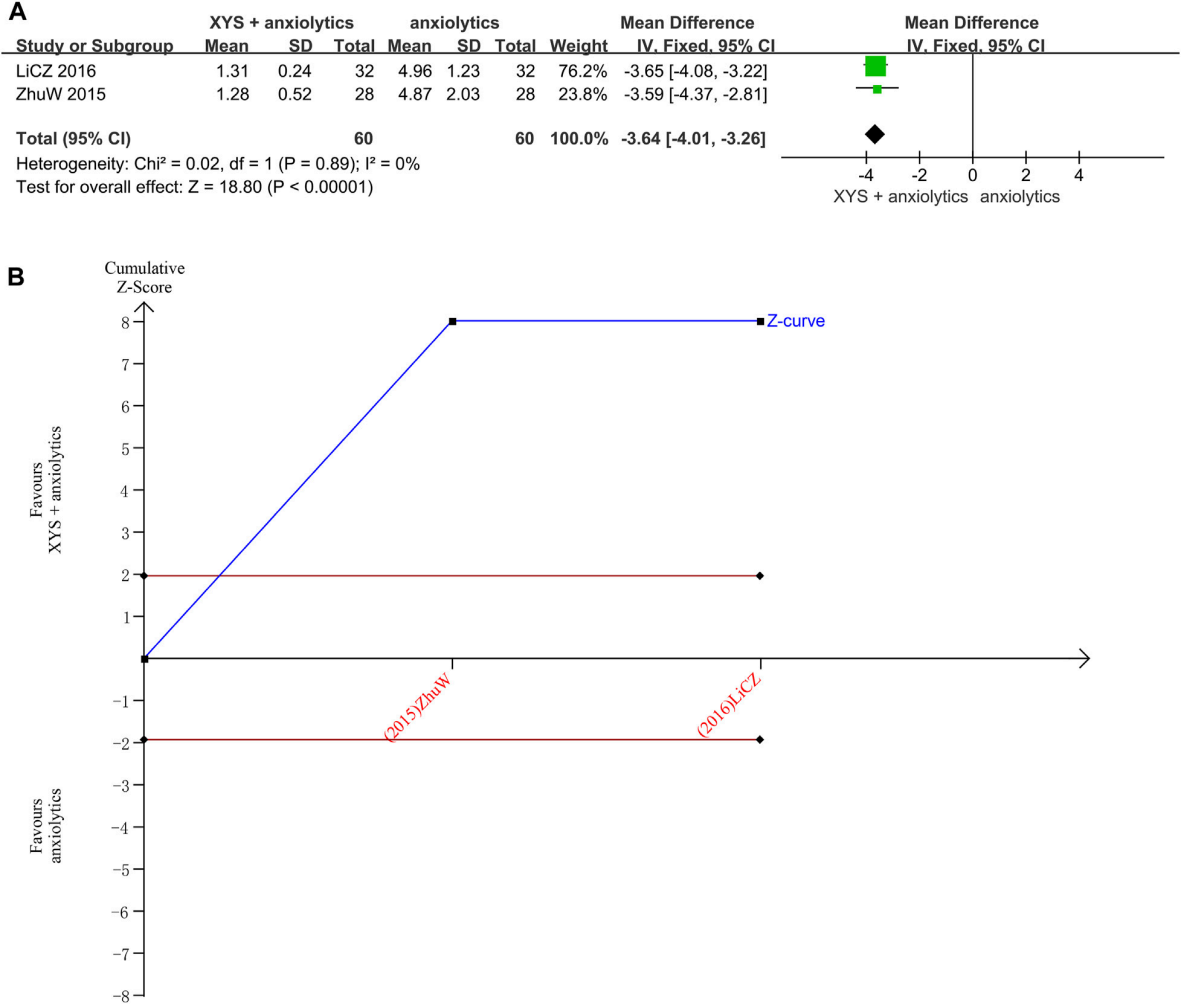
Data analysis of TESS scores (XYS combined with anxiolytics vs. anxiolytics alone). **(A)** Meta-analysis. **(B)** Trial sequential analysis. The annotations of the red line and blue line in TSA are the same as those in Figure 3.

Two studies reported the TESS scores (Zhu, 2015; LI and Qin, 2016). No heterogeneity was detected when these data were analyzed (*p* = 0.89, I^2^ = 0%). The results were thus analyzed using a fixed-effects model. The pooled analysis results indicated that the TESS scores in the treatment group were significantly lower than those in the control group ([MD = 3.64, 95% CI: −4.01, −3.26, *p* < 0.00001]; Figure 7A).

The TSA analysis for the TESS scores showed that the cumulative Z-curve exceeded the traditional significance boundary, but the sequential monitoring boundary could not be rendered because the first information fraction exceeded 100%, which indicated that the first study showed sufficient statistical power for a meta-analysis. The results of the TSA suggested that compared to treatment with anxiolytics, treatment with XYS combined with anxiolytics significantly decreased the TESS score of patients with anxiety symptoms; the results were stable, and the evidence was reliable (Figure 7B).

#### 3.3.2 Comparison of treatment with XYS alone vs. anxiolytics alone

##### 3.3.2.1 Clinical efficacy rates

**FIGURE 8:**
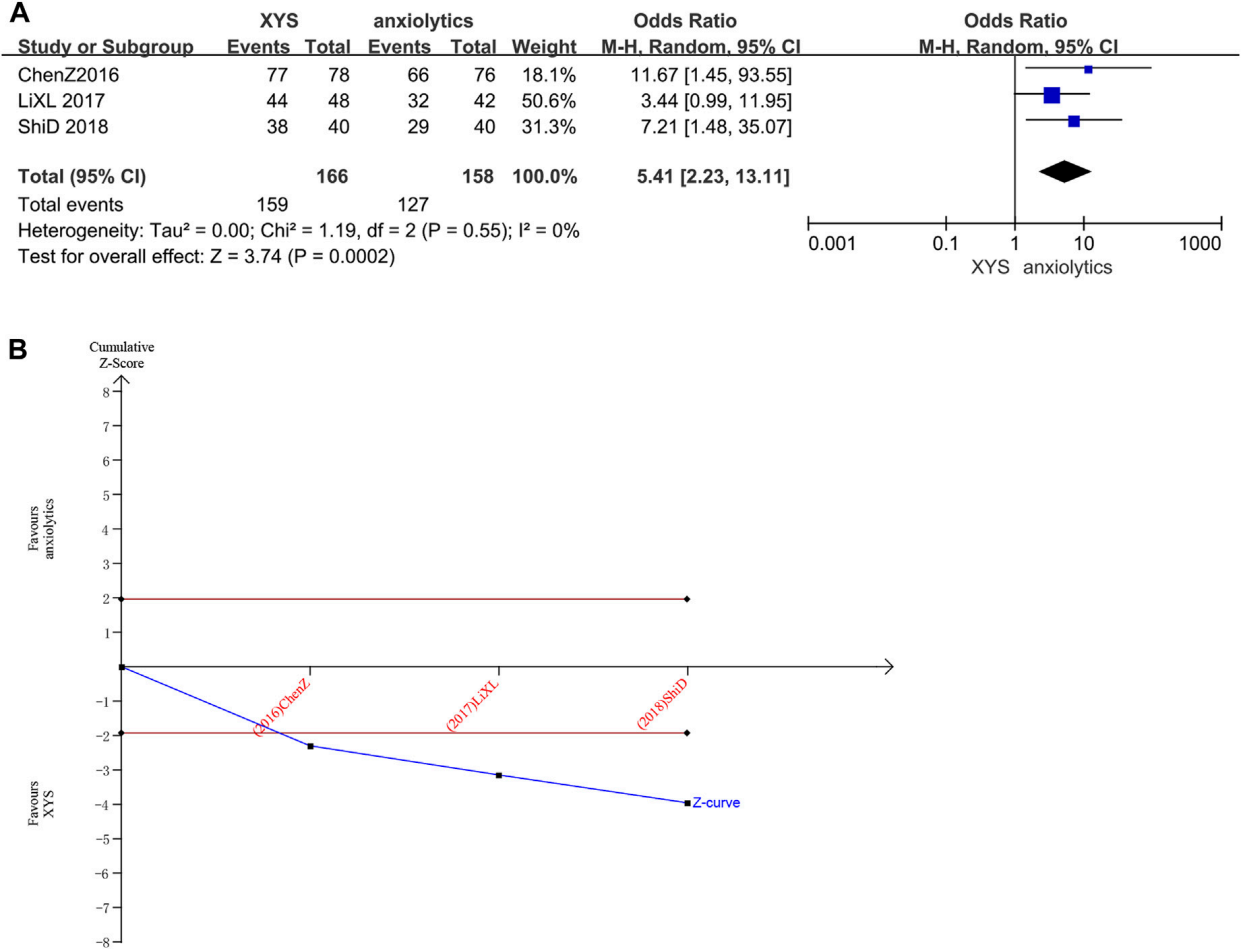
Data analysis of the clinical efficacy rates (XYS vs. anxiolytics). **(A)** Meta-analysis. **(B)** Trial sequential analysis. The annotations of the red line and blue line in TSA are the same as those in Figure 3.

Three studies (*n* = 324 patients) (Chen and Du, 2016; Li et al., 2017; Shi, 2018) reported clinical efficacy rates. Several studies did not significantly differ in clinical efficacy rates from one another (Chi^2^ = 1.19, *p* = 0.55, I^2^ = 0%). The efficacy recorded in the treatment groups was substantially higher than that recorded in the control groups, as determined by the results of the random-effects model (RR = 5.41, 95% CI: [2.23, 13.11], *p* < 0.0001, Figure 8A).

The cumulative Z-curve for Clinical Efficacy Rates scores exceeded the conventional significance boundary, as determined by the results of the TSA analysis, but the sequential monitoring boundary could not be rendered because the first information fraction was greater than 100%, which indicated that the initial study obtained enough statistical power for a meta-analysis. The results of the TSA matched those of the meta-analysis, suggesting that compared to treatment with anxiolytics, treatment with XYS can significantly improve the anxiety symptoms of patients with emotional disorders; the results were stable, and the evidence was reliable (Figure 8B).

##### 3.3.2.2 HAMA scores

**FIGURE 9:**
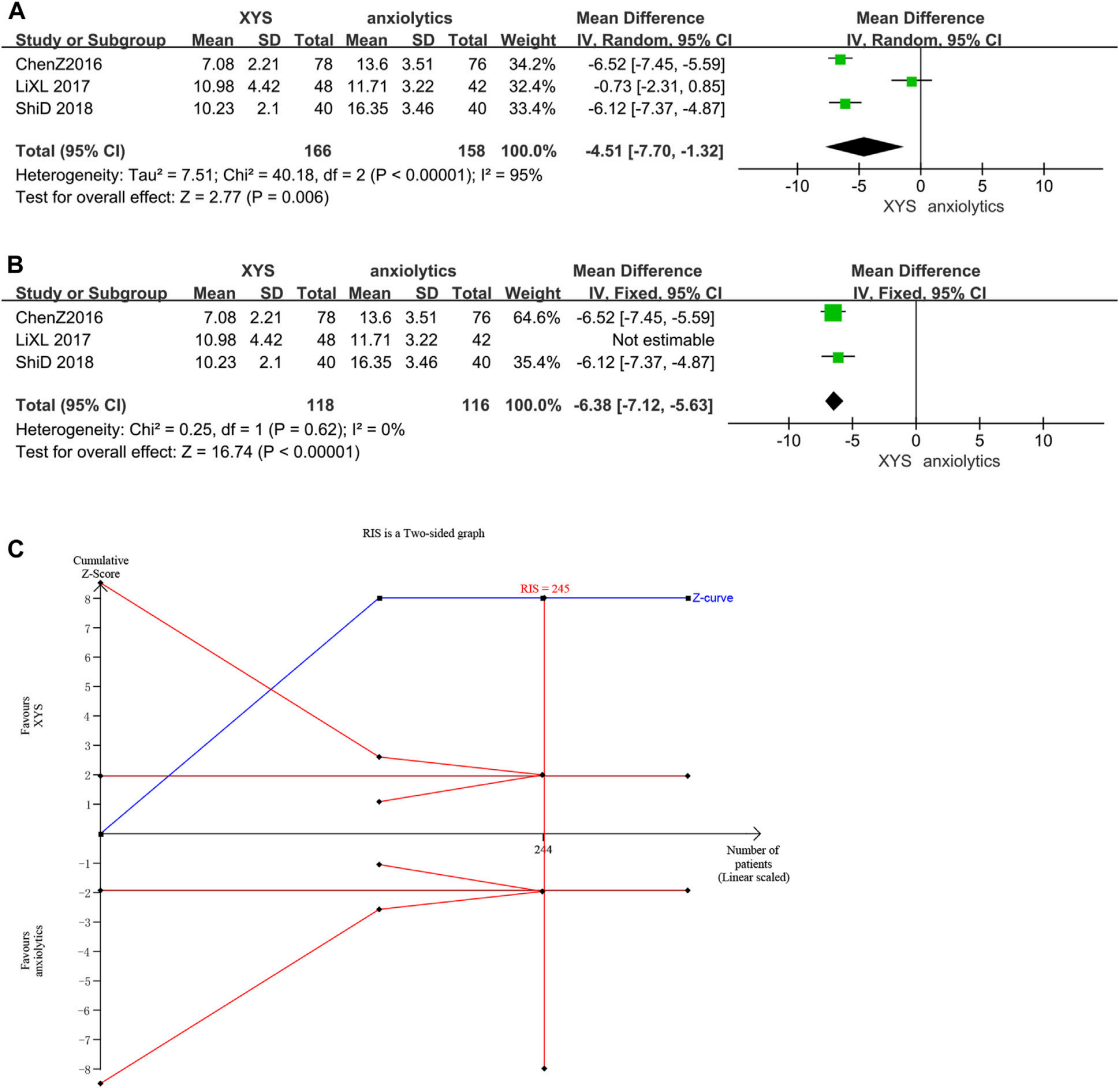
Data analysis of HAMA-14 scores (XYS vs. anxiolytics). **(A)** Before eliminating sources of heterogeneity. **(B)** After eliminating sources of heterogeneity. **(C)** Trial sequential analysis. The annotations of the red line and blue line in TSA are the same as those in Figure 3.

Three studies (Chen and Du, 2016; Li et al., 2017; Shi, 2018) reported the HAMA scores, and there was significant variation among these studies (*p* < 0.00001, I^2^ = 95%). The HAMA scores in the treatment group were considerably lower than those in the control group ([MD = −4.51, 95% CI: −7.70, −1.32, *p* = 0.006]; Figure 9A), as determined by the results of a random-effects meta-analysis. According to the analysis of the treatment course, the source of heterogeneity was one study (Li et al., 2017). In this study, Lorazepam was used in the anxiolytics group, whereas other studies used Deanxit. A meta-analysis of the other two studies showed that XYS therapy reduced the HAMA scores of AD patients with low heterogeneity and was more effective than anxiolytic therapy ([MD = −6.38, 95% CI: −7.12, −5.63]; *p* < 0.00001; I^2^ = 0%; Figure 9B).

The first information fraction was greater than 100%, which indicated that the initial study had sufficient statistical power for a meta-analysis, and the cumulative Z-curve for the HAMA scores exceeded the conventional significance boundary, as determined by the TSA analysis. The TSA suggested that compared to anxiolytics, XYS significantly decreased the HAMA score of patients with anxiety symptoms; the results were stable, and the evidence was reliable (Figure 9C).

##### 3.3.2.3 SAS scores

The SAS scores were reported in one study (Shi, 2018). As trial sequence analysis and meta-analysis could not be performed, descriptive analysis was conducted. The findings showed that the XYS group had significantly lower SAS scores than the anxiolytics group ([MD = −13.50, 95% CI: −15.26, −11.74]), *p* < 0.00001).

##### 3.3.2.4 TESS scores and adverse events rates

The Adverse Events Rates and TESS scores were reported in one study (Li et al., 2017). As trial sequence analysis and meta-analysis could not be performed, descriptive analysis was conducted instead. Our findings showed that the XYS group had considerably lower TESS ratings than the anxiolytics group ([MD = −1.22, 95% CI: −1.82, −0.62]), *p* < 0.0001). The adverse events of the XYS group were not significantly different from those of the other group ([RR = 0.29, 95% CI: 0.06, 1.37, *p* = 0.12]).

## 4 Discussion

In this study, we provided new information for therapeutic decision-making by conducting a meta-analysis of the randomized controlled trial of XYS in treating anxiety disorder and by conducting a more impartial evaluation of this study through TSA. The TSA we performed on the efficacy of XYS for managing anxiety showed that the cumulative sample size was adequate to support the meta-analysis. The TSA, however, could not correct mistakes that occurred due to methodological flaws in the included RCTs. As the sometimes subpar quality of RCTs might have affected the reliability of TSA outcomes, the conclusions must be interpreted with caution.

In this study, 14 randomized controlled trials were included; 11 studies on XYS combined with anti-anxiety Western medicine and three studies on XYS compared to Western medicine. When XYS was compared to anti-anxiety medication and XYS alone for evaluating the effective rate and the HAMA score, the results were more favorable for the former, indicating that XYS can be used as an alternative or supplemental treatment for anxiety disorders.

The adverse event rates in the XYS combined with the anxiolytics group were significantly lower than those in the anxiolytics group, whereas no significant difference was found between the adverse events of the XYS group and the anxiolytics group, although the incidence of adverse events in the XYS group was lower than that in the anxiolytics group. TSA of the XYS combined with anxiolytics vs. anxiolytics showed that the adverse event rates reached the expected sample size.

We also analyzed secondary indicators, including the SAS score, PSQI score, and TESS score. The SAS score was reported in only one study on XYS combined with anxiolytics treatment and anxiolytics alone treatment. Two studies reported the PSQI scores for the XYS combined with the anxiolytics group and the anxiolytics group. Two studies reported the TESS scores for the XYS combined with the anxiolytics group vs. the anxiolytics group, and one study reported the TESS scores for the XYS group vs. the anxiolytics group. These findings led to more positive results. However, the results need to be interpreted with caution as the sample size was small.

Anxiety is an extremely common mental health problem, and it can adversely affect daily life and general wellbeing. Anxiety may occur with or contribute to the development or worsening of medical conditions, including cardiovascular diseases, gastrointestinal diseases, pulmonary diseases, cancer, chronic pain, and migraine headaches (Hackbarth et al., 1986). Oliver et al. speculated that the bed nucleus of the stria terminalis, the amygdala, and the hippocampus, and their connection to the cortical areas like the dorsal medial and lateral prefrontal/cingulate cortex and the insula, plays an important role in maintaining anxiety response (Robinson et al., 2019). The most commonly prescribed anti-anxiety medications are quetiazide, venlafaxine, paroxetine, escitalopram, and duloxetine. These medications have limited tolerance but good remission effects (Kong et al., 2020).

According to TCM, emotional elements directly affect the etiology of anxiety. Emotional disorders can lead to stagnation of the liver qi, dysfunction of spleen transport, and disorder of qi and blood, which is known as liver stagnation and spleen deficiency syndrome (LSSDS). Throughout treatment, the fundamental therapeutic effects of TCM include nourishing blood, tonifying the spleen, and soothing liver-qi stagnation. XYS can soothe the liver, relieve depression, nourish the blood, and invigorate the spleen. For over a century, XYS has been used in TCM clinics to treat various illnesses that share the symptoms of liver stagnation and spleen deficiency syndrome (LSSDS). A component of XYS known as Chai Hu is used to spread stagnant liver qi and treat depression. It also regulates liver qi, making it an essential monarch drug. Bai Shao, Dang Gui, and stewed ginger, which are used as ministerial drugs, can nourish the blood. Astringe yin can nourish the liver and reduce stress. As adjuvants, Bai Zhu, Fu Ling, and licorice can energize the spleen, remove moisture, facilitate transportation and transformation, and provide qi and blood to their source. A small amount of mint is used as a conductive medicine, which helps inhibit the spread of qi and reach the liver meridian to treat the syndrome of heat stagnation. Suitable medications can be administered to treat the liver and spleen while also nourishing the liver and promoting liver function.

The mechanism of the anti-anxiety effect of XYS was found in some animal experiments. Hao et al. found that XYS can reduce anxiety-like behavior by controlling the gut microbiota, reducing excessive LPS production, and preventing excessive activation of NLRP3 inflammasome in the colon (Hao et al., 2021). XYS can limit the expression of miR-200a/b-3p that is produced by CUMS, control miR-200a/b-3p/NR3C1 signaling in the prefrontal cortex that is induced by chronic stress, and decrease neuronal death and anxiety-like behavior (Yuan et al., 2020). In an animal study, it was found that XYS can promote the regeneration of hippocampal neurons by blocking the Notch signal pathway, which decreases anxiety-provoking behavior (Liu et al., 2021). Zhao et al. showed that XYS can maintain mitochondrial function by increasing the level of BDNF and phosphorylated AMPK, thus alleviating anxiety (Zhao et al., 2022). XYS can treat anxiety and depression caused by a high-fat diet by regulating metabolites derived from the intestinal microflora (Yang et al., 2022).

## 5 Limitations and recommendations

Our study had some limitations. First, poor quality of methodology is a common problem in studies on traditional Chinese medicine. The studies included also lacked a detailed description of random methods, random assignment concealment, blind method implementation, selective outcome reports, etc. The randomization method only describes the representation of random numbers and is ambiguous. In most cases, randomization is employed to balance known and unknown confounding factors between experimental and control groups. It is recommended that future studies provide detailed descriptions of the randomization methods used, such as simple randomization, block randomization, stratified randomization, and cluster randomization. To prevent the knowledge of the randomization results from biasing the researchers, it is preferable to implement concealed randomization using methods such as sealed envelopes or central randomization. Central randomization is suitable for large multicenter studies, while sealed envelopes are applicable to small-sample clinical studies conducted at a single center. Additionally, implementing blinding procedures is also crucial. It is hoped that future studies can achieve blinding of participants, observers, and outcome assessors/data analysts. Second, due to the limitations of traditional Chinese medicine, implementing a blind method is difficult. Double-blind and double-simulation designs may be used in future studies on traditional Chinese medicine. Most of the included studies were single-center, small-sample studies with low-level evidence. More high-quality evidence with more participants and from multiple centers is needed. Third, studies included in this manuscript lacked a long-term follow-up evaluation, and evaluating the long-term efficacy of XYS was difficult. Finally, all participants were Chinese, and the applicability of the conclusions of the study to individuals of other nationalities is limited. XYS has been registered in Denmark and is used for alternative self-care of European patients to relieve mental stress. This provides a basis for research on the applicability of XYS to individuals of other nationalities. We strongly recommend conducting multi-center, double-blind, and double-simulated high-quality clinical studies to obtain more detailed information and reliable results.

## 6 Conclusion

To assess the effectiveness and safety of administering XYS for treating anxiety disorder, we conducted a meta-analysis and trial sequential analysis. We found that XYS can be used safely and effectively to treat anxiety combined with Western medicine. However, although XYS alone was found to be effective in treating anxiety disorder, more large-scale studies are needed to determine adverse events. Additionally, considering that the methodology had low quality, the results should be interpreted with caution.

## Data Availability

The original contributions presented in the study are included in the article/Supplementary Material, further inquiries can be directed to the corresponding authors.
