# Supplementary material for: Efficacy and safety of the Chinese herbal medicine Xiao Yao San for treating anxiety: a systematic review with meta-analysis and trial sequential analysis
Source: Front Pharmacol. 2023 Oct 12;14:1169292. doi: 10.3389/fphar.2023.1169292 (PMC10613521; doi:10.3389/fphar.2023.1169292)
Supplement: Supplementary file 1 [file DataSheet1.docx]

Supplementary Material

Efficacy and safety of Chinese herbal medicine Xiao Yao San in anxiety: A Systematic Review with Meta-Analysis and Trial Sequential Analysis

Yifan Wang ^1^ ^†^, Xiaofeng Chen^1^ ^†^, Wei Wei^1^, Yiyun Ding^2^, Rongjuan Guo^2^*, Jia Xing^2*^, Jialin Wang^3*^

*** Correspondence:** Rongjuan Guo, dfguorongjuan@163.com; Jia Xing, dfyyxingjia@163.com; Jialin Wang, wjl2008420@163.com

^†^**These authors contributed equally to this work and share first authorship**

# The search strategy

**Search run on Thurs August 10 2023**

**MEDLINE(Ovid) n=25**

Ovid MEDLINE(R) ALL <1946 to August 08, 2023>

1 exp Anxiety/

2 exp Anxiety Disorders/

3 (angst or social anxiet* or anxiet* or hypervigilance or nervousness or anxiousness).af.

4 1 or 2 or 3

5 (xiaoyao* or xiao yao* or xiao-yao* or Kami-shoyo-san or Gamisoyo-san or soyo-san or Kamo-soyo-san).af.

6 exp Randomized Controlled Trial/

7 exp Controlled Clinical Trial/

8 (random* or clinical trial or trial).af.

9 6 or 7 or 8

10 4 and 5 and 9

**Embase n=33**

#1. 'anxiety'/exp

#2. 'anxiety disorders'/exp

#3. 'angstrom' OR 'social anxiet*' OR 'anxiet*' OR 'hypervigilance' OR 'nervousness' OR 'anxiousness'

#4. #1 OR #2 OR #3

#5. 'xiaoyao*' OR 'xiao yao*' OR 'xiao-yao*' OR 'Kami-shoyo-san' OR 'Gamisoyo-san' OR 'soyo-san' OR 'Kamo-soyo-san'

#6. 'randomized controlled trial'/exp

#7. 'controlled clinical trial'/exp

#8. 'random' OR 'clinical trial' OR 'trial'

#9. #6 OR #7 OR #8

#10. #4 AND #5 AND #9

**Cochrane library n=36**

#1 MeSH descriptor: [Anxiety] explode all trees

#2 MeSH descriptor: [Anxiety Disorders] explode all trees

#3 (angst or (social anxiet*) or anxiet* or hypervigilance or nervousness or anxiousness)

#4 #1 or #2 or #3

#5 (xiaoyao*) or (xiao yao*) or (xiao-yao*) or (*shoyo-san) or (*soyo-san) or (Kami-shoyo-san) or (Gamisoyo-san) or (Kamo-shoyo-san)

#6 #4 and #5

#7 MeSH descriptor: [Randomized Controlled Trial] explode all trees

#8 MeSH descriptor: [Controlled Clinical Trial] explode all trees

#9 (random* or (clinical trial) or trial)

#10 #7 or #8 or #9

#11 #6 and #10

**Psycinfo n=9**

1 MESH(anxiety) or MESH(anxiety disorders) or ALL(angst or social anxiet* or anxiet* or hypervigilance or nervousness or anxiousness)

2 ALL(xiaoyao* or (xiao yao*) or xiao-yao* or "Kami-shoyo-san" or "Gamisoyo-san" or "soyo-san") or "kamo-soyo-san"

3 MESH(Randomized Controlled Trial) or MESH(Controlled Clinical Trial) or ALL(random* or (clinical trial) or trial)

4 #1 and #2 and #3

**The Allied and Complementary Medicine Database（AMED） n=55**

S1:SU Anxiety OR SU Anxiety Disorders OR TX angst OR TX social anxiet*) OR TX anxiet* OR TX hypervigilance OR TX nervousness OR TX anxiousness

S2:TX xiaoyao* OR TX xiao yao* OR TX xiao-yao* OR TX *shoyo-san OR *soyo-san

S3:SU Randomized Controlled Trial OR SU Controlled Clinical Trial OR TX random OR TX clinical trial OR TX trial

S4:S1 AND S2 AND S3

**PubMed n=27**

1 (((((((Anxiety[MeSH Terms]) OR (Anxiety Disorders[MeSH Terms])) OR (angst)) OR (social anxiet*)) OR (anxiet*)) OR (hypervigilance)) OR (nervousness)) OR (anxiousness)

2 ((((((xiaoyao*) OR (xiao yao*)) OR (xiao-yao)) OR (Kami-shoyo-san)) OR (Gamisoyo-san)) OR (soyo-san)) OR (Kamo-shoyo-san)

3 ((((Randomized Controlled Trial[MeSH Terms]) OR (Controlled Clinical Trial[MeSH Terms])) OR (random*)) OR (clinical trial)) OR (trial)

4 #1 and #2 and #3

**Science Citation Index Expanded (Web of Science) n=48**

1: (((((((TS=(Anxiety)) OR TS=(Anxiety Disorders)) OR ALL=(angst)) OR ALL =(social anxiet*)) OR ALL =(anxiet*)) OR ALL =(hypervigilance)) OR ALL =(nervousness)) OR ALL =(anxiousness)

2: (((ALL=(xiaoyao*)) OR ALL =(xiao yao*)) OR ALL =(xiao-yao*)) OR ALL =(Kami-shoyo-san) OR ALL =( Gamisoyo-san) OR ALL =(soyo-san) OR ALL =(Kamo-soyo-san)

3: ((((TS=(randomized controlled trial)) OR TS=(controlled clinical trial)) OR ALL=(random*)) OR ALL=(clinical trial)) OR ALL=(trial)

4: #1 AND #2 AND #3 48

**Scopus n=106**

#1 TITLE-ABS-KEY ( "Anxiety" OR "Anxiety Disorders" ) OR ALL ( "angst" OR "social anxiet*" OR "anxiet*" OR "hypervigilance" OR "nervousness" OR "anxiousness" )

#2 TITLE-ABS-KEY ( "xiaoyao*" OR "xiao yao*" OR "xiao-yao*" OR "*shoyo-san" OR "*soyo-san" )

#3 TITLE-ABS-KEY ( "randomized controlled trial" OR "controlled clinical trial" OR random* OR "clinical trial" OR trial )

#4 #1 and #2 and #3

**SinoMed西文数据库 n=6**

1 "Anxiety"[常用字段:智能] OR "Anxiety Disorder"[常用字段:智能] OR "angst"[全部字段:智能] OR "social anxiet*"[全部字段:智能] OR "anxiet*"[全部字段:智能] OR "hypervigilance"[全部字段:智能] OR "nervousness"[全部字段:智能] OR "anxiousness"[全部字段:智能] 315065

2 "xiaoyao*"[全部字段:智能] OR "xiao yao*"[全部字段:智能] OR "xiao-yao*"[全部字段:智能] OR "*shoyo-san"[全部字段:智能] OR "*soyo-san"[全部字段:智能]

3 "Randomized Controlled Trial"[常用字段:智能] OR "Controlled Clinical Trial"[常用字段:智能] OR "random"[全部字段:智能] OR "clinical trial"[常用字段:智能] OR "trial"[常用字段:智能] 902657

4 (#1) AND (#2) AND (#3)

**SinoMed中文数据库 n=256**

1 "焦虑"[常用字段:智能] OR "焦虑*"[常用字段:智能] OR "焦虑障碍"[常用字段:智能] OR "焦虑症"[常用字段:智能]

2 "逍遥散"[常用字段:智能] OR "逍遥汤"[常用字段:智能] OR "逍遥片"[常用字段:智能] OR "逍遥颗粒"[常用字段:智能] OR "逍遥胶囊"[常用字段:智能] OR "逍遥*"[常用字段:智能]

3 "随机对照试验"[常用字段:智能] OR "临床对照试验"[常用字段:智能] OR "随机"[全部字段:智能] OR "临床研究"[常用字段:智能] OR "临床试验"[常用字段:智能]

4 (#1) AND (#2) AND (#3)

**中国知网CNKI n=466**

1 TKA= '焦虑' + '焦虑障碍' + '焦虑症' + '焦虑*'

2 TKA= '逍遥散' + '逍遥汤' + '逍遥片' + '逍遥胶囊' + '逍遥颗粒' + '逍遥*'

3 TKA= '随机对照试验' + '临床对照试验' + '临床研究' + '临床试验' or FT=随机

4 #1 and #2 and #3

**万方 n=368**

1 主题:(焦虑) or 主题:(焦虑障碍) or 主题:(焦虑症) or 主题:(焦虑*)

2 主题:(逍遥散) or 主题:(逍遥汤) or 主题:(逍遥片) or 主题:(逍遥胶囊) or 主题:(逍遥颗粒) or 主题:(逍遥*)

3 主题:(随机对照试验) or 主题:(临床对照试验) or 主题:(临床研究) or 主题:(临床试验) or 全部:(随机)

4 #1 and #2 and #3

**维普 n=86**

M=("焦虑" OR "焦虑障碍" OR "焦虑症" OR "焦虑*") AND M=("逍遥散" OR "逍遥汤" OR "逍遥片" OR "逍遥胶囊" OR "逍遥颗粒" OR "逍遥*") AND (M=("随机对照试验" OR "临床对照试验" OR "临床研究" OR "临床试验") OR U="随机")

# Supplementary Figures and Tables

## Supplementary Figures


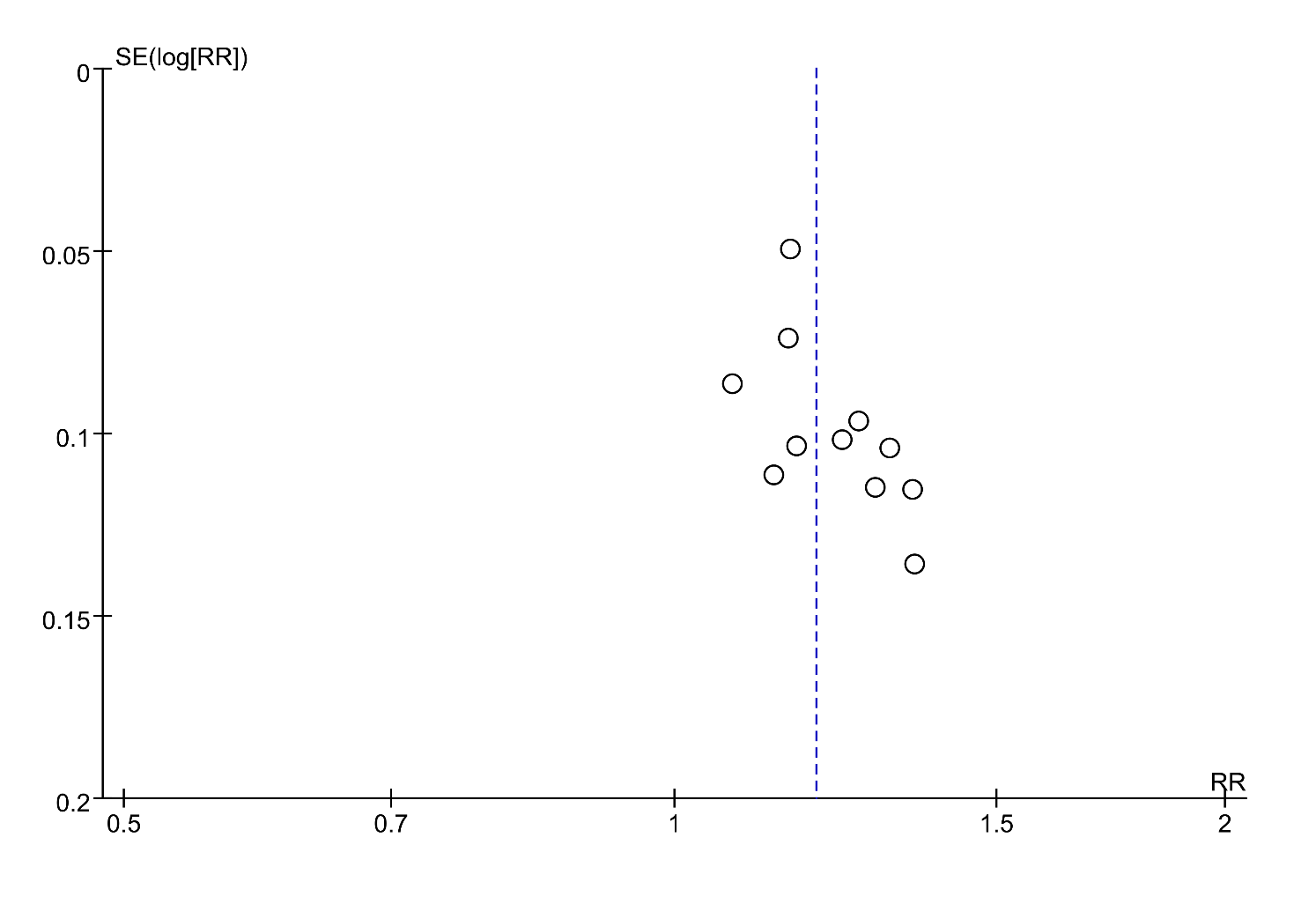


**Figure S1. Funnel plot: Data analysis of clinical efficacy rates (XYS + anxiolytics vs. anxiolytics Alone)**


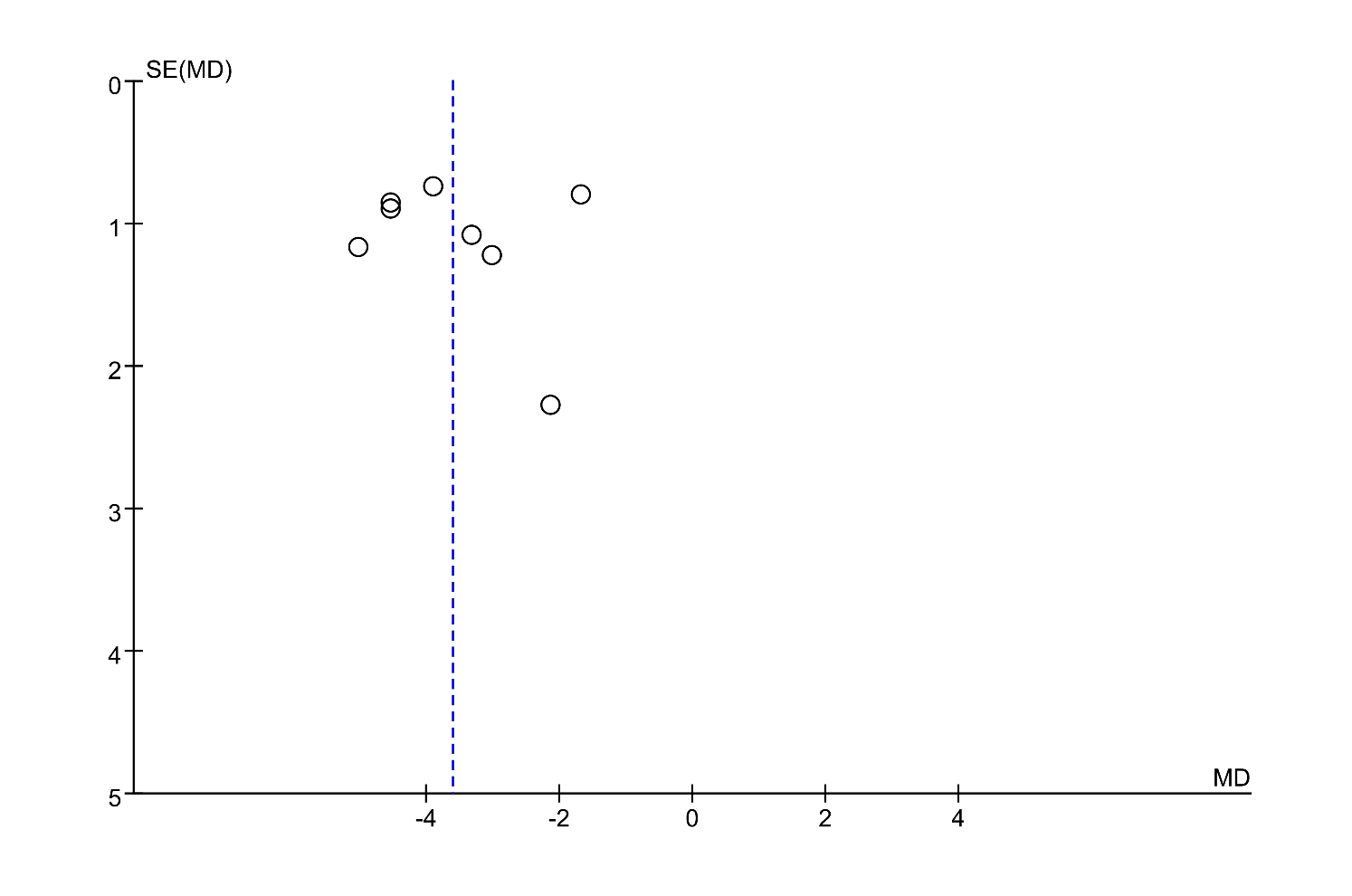


**Figure S2. Funnel plot:** **Data analysis of HAM-A Scores (XYS + anxiolytics vs. anxiolytics Alone)**
